# Group versus Individual Acupuncture (AP) for Cancer Pain: A Randomized Noninferiority Trial

**DOI:** 10.1155/2020/7209548

**Published:** 2020-04-13

**Authors:** Erica Nicole Reed, Jessa Landmann, Devesh Oberoi, Katherine-Ann L. Piedalue, Peter Faris, Linda E. Carlson

**Affiliations:** ^1^Division of Psychosocial Oncology, Department of Oncology, Cumming School of Medicine, University of Calgary, Calgary, AB, Canada; ^2^Vive Integrative Health Group, 1889 45 Street NW, Calgary, AB, Canada; ^3^Department of Analytics, Alberta Health Services (AHS) and Department of Community Health Sciences, University of Calgary, Calgary, AB, Canada

## Abstract

**Background:**

A service delivery model using group acupuncture (AP) may be more cost-effective than individual AP in general, but there is little evidence to assess whether group AP is a comparable treatment in terms of efficacy to standard individual AP. The study aimed to compare the group to individual delivery of 6-week AP among cancer patients with pain.

**Methods:**

The study design was a randomized noninferiority trial of the individual (gold standard treatment) vs. group AP for cancer pain. The primary outcome was pain interference and severity, measured through the Brief Pain Inventory (BPI). Secondary outcomes included measures of mood, sleep, fatigue, and social support. Changes in outcomes from pre- to postintervention were examined using linear mixed effects modeling and noninferiority was inferred using a noninferiority margin, a difference of change between the two arms and 95% CIs. Pain interference was tested with a noninferiority margin of 1 on the BPI, while pain severity and secondary outcomes were compared using conventional statistical methods.

**Results:**

The trial included 74 participants randomly allocated to group (35) or individual (39) AP. The noninferiority hypothesis was supported for pain interference [Ө − 1, Δ 1.03, 95% CI: 0.15–2.20] and severity [Ө − 0.81, Δ 0.52, 95% CI:.33–1.38] as well as for mood [Ө − 7.52, Δ 9.86, 95% CI: 0.85–18.86], sleep [Ө − 1.65, Δ 2.60, 95% CI: 0.33–4.88], fatigue [Ө 8.54, Δ − 15.57, 95% CI: 25.60–5.54], and social support [Ө.26, Δ − 0.15, 95% CI:  − 0.42–0.13], meaning that group AP was not inferior to individual AP treatment. Both arms evidenced statistically significant improvements across all symptoms before and after the intervention. Effect sizes for the group vs. individual AP on outcomes of pain, sleep, mood, and social support ranged from small to very large and were consistently larger in the group condition. The total average cost-per-person for group AP ($221.25) was almost half that of individual AP ($420).

**Conclusions:**

This is the first study to examine the noninferiority of group AP with the gold standard individual AP. Group AP was noninferior to individual AP for treating cancer pain and was superior in many health outcomes. Group AP is more cost-effective for alleviating cancer pain and should be considered for implementation trials.

## 1. Background

As the number of cancer survivors continues to grow, complementary therapies have become increasingly more relevant to help treat many symptoms caused by cancer and its therapies. Pain is one of the most commonly reported symptoms, with a review of 122 studies showing that pain conditions were experienced by 39.3% of patients after curative treatment, 55% of those on active treatment, and 66.4% of patients with advanced, metastatic, or terminal cancer [1]. Pain is both extremely common, as well as undertreated, with up to 43% of people with cancer pain reporting not having sufficient pain treatment [2]. While opioids are commonly used to treat cancer pain, they often come with a range of side effects including nausea, constipation, and drowsiness [3]. Further, in the midst of the opioid crisis, patients and providers are seeking alternatives to avoid opioid overuse and abuse in the treatment of cancer pain [4].

Acupuncture (AP) is a therapeutic technique derived from Traditional Chinese Medicine (TCM) and is one complementary therapy modality that has been used for treating pain. Previous studies have supported the analgesic effects of AP both within the general population and specifically for cancer patients [5, 6]. A systematic review of the oncology-AP literature concluded that research supports using AP to alleviate various cancer-related symptoms, including hot flashes, nausea and vomiting, pain, and fatigue [7]. Among these, the evidence is strongest for the use of AP for cancer pain management [8, 9]. Along with its effectiveness for managing pain, the relatively low risk of side effects and the low number of adverse events make it an appealing treatment option for patients.

In North America, AP is typically performed on an individual basis, and not often covered by insurance, which may be cost-prohibitive to some patients. The cost for AP treatments has been reported as increasing at a faster rate than inflation, making these treatments less accessible for people in lower socioeconomic statuses [10]. Thus, it is important to investigate alternative delivery methods of AP that may help reduce the cost of treatments. Group (or community) AP is an emerging cost-effective method of treating patients. Although the practice can vary, group AP is performed in a group setting, typically with reclining chairs dispersed around a large room, and one practitioner treating up to six people per session [11, 12]. Despite its growing popularity, there is relatively little evidence to demonstrate the effectiveness of AP performed in a group setting. One retrospective analysis suggested that group AP was helpful in reducing cancer-related pain, numbness, and digestive problems after four sessions, and another trial of otherwise healthy individuals found that after 24 weeks of treatment with group AP, primary care patients with chronic pain experienced decreases in both pain severity and inference [13, 14]. In the only randomized controlled trial (RCT) that has investigated this novel treatment, group AP was compared to education sessions for women with fibromyalgia; participants who received group AP experienced improvements in pain and fatigue levels when compared to the participants who only received education [15].

To date, there have been no published RCTs that have directly compared group AP to typical, individual AP. Without this knowledge, it is impossible to assess whether administering AP in a group setting is a comparable treatment to the standard individual AP for treating pain. This information is relevant because, in addition to its possible effectiveness at reducing pain, group AP has also been reported as a source of social support to patients, as well as having the potential to be more cost-effective than individual treatment [16, 17]. This potential for cost-savings is important, not only because we want to reach patients of lesser means, but also because AP is not a treatment that lasts indefinitely; for ongoing pain control, patients must undergo AP on a regular basis until they start seeing benefits, and this likely requires periodical booster sessions. Unfortunately, the high cost per treatment of individual AP may prevent patients from attending sessions as often as would be optimal. Thus, it would be ideal to find a way to administer AP at a lower cost, especially if the potential exists to provide other benefits concomitantly, such as social support, which has previously been associated with increased emotional well-being in the cancer population [18].

Therefore, in this randomized noninferiority trial, we aimed to compare the group to individual delivery of 6-week AP program for alleviating cancer pain. The specific objectives of the study were as follows:

1. To compare the group to individual AP on pain interference and severity as well as on sleep, distress, fatigue, and perceived social support
2. To compare the costs associated with the group and individual AP

### 1.1. Hypotheses

1. Improvements in sleep disturbance, distress, and fatigue as well as social support from baseline to postintervention among participants receiving group AP will be noninferior to individual delivery
2. Group AP will be associated with lower costs compared to individual AP

## 2. Methods

### 2.1. Study Design

This study was a two-group noninferiority randomized controlled trial, with participants being randomized to receive either group or individual AP. The noninferiority trial design was chosen because our primary research question was whether group AP was inferior to the gold standard treatment for pain, individual AP, or not.

### 2.2. Inclusion and Exclusion Criteria

**Figure 1:**
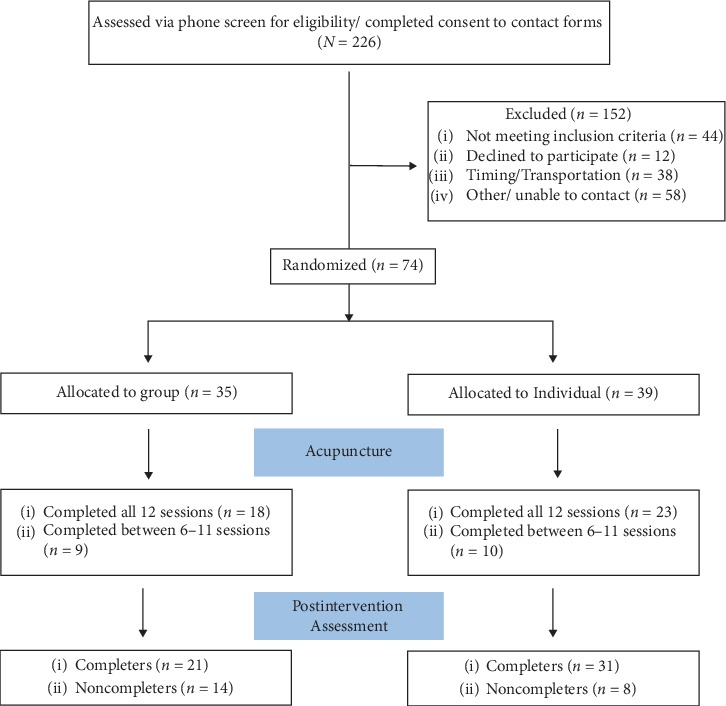
CONSORT flowchart.

Inclusion criteria were meant to be pragmatic and included the following: (1) male or female cancer patients, (2) ≥18 years old, (3) all tumor groups, (4) metastatic or nonmetastatic, (5) experiencing pain with a minimum worst pain score (in the previous week) ≥3 on the 10-point Brief Pain Inventory (BPI), (6) willing to be randomized to either condition and lastly, and (7) able to attend a minimum of 9 treatment sessions within a 6-week period. Exclusion criteria included the following: (1) AP treatments within the previous six months (as it could either underestimate or inflate the effects of the intervention) and (2) a change in cancer treatment that may affect pain control (radiation, systemic therapy, and surgery) within the previous six weeks (Figure 1). This was because these treatments may cause rapid changes in health status, and the completion of treatment may result in spontaneous improvement of symptoms, therefore confounding the results.

### 2.3. Participants and Procedures

Participants were recruited through various outpatient oncology clinics at the Tom Baker Cancer Centre in Calgary, Alberta, Canada. “Consent to Contact” forms were attached to patient charts, and brochures with information about the study were placed at these centers, allowing participants to self-refer to the study. Those who self-referred or filled out consent to contact forms were screened for eligibility, and all those determined eligible were invited to participate in the study. Those who agreed to participate visited the cancer center to fill out baseline questionnaires and meet one-on-one with the acupuncturist for an intake assessment. After this initial assessment, participants were randomized to either the group or individual AP treatments, using block randomization, which was stratified by sex. Treatments for both arms took place twice weekly over the course of six weeks, for a total of twelve treatments. All AP sessions were provided to participants at no cost. Any major changes in health, possible adverse events, and general well-being were assessed by research assistants during each week of the intervention.

### 2.4. Treatments

An accredited Naturopathic Medicine Practitioner (JL) performed treatment in both groups of participants. At the time of the study, she had been in practice for four years, specifically working in the oncology population.

#### 2.4.1. Individual AP

AP treatments were based on TCM theory and previous research protocols. Because this was meant to be a pragmatic trial, protocols were individualized, not standardized. The only standardized acupoints were LI4 and LV3, points commonly used for pain. Treatments were open to modification over the course of the study to accommodate the individual's changing symptomatology. The needles used were TeWa, noncoated, stainless steel disposable needles (0.25  mm × 40  mm). The depth of insertion was based on when the participant experienced *De qi* sensation. Participants received 12 to 22 needles per session. After 10–15 minutes of needle retention, the practitioner manually manipulated them in order to elicit the *De qi* sensation. Needles were retained for 20 to 25 minutes. The total appointment time was 45 minutes.

#### 2.4.2. Group AP

Group AP was performed in a room that accommodated up to 6 people per session. Participants were given a 30-minute window to arrive, and treatments were performed on a first-come-first-served basis. Participants were seated comfortably in reclining chairs spaced in a large circle. Participants were instructed that during the session, they were free to either sit quietly or to talk among themselves. Identical needle placement and manipulation procedures to the individual treatments were followed. Each group session was scheduled for 90 minutes to allow for latecomers, with each participant staying for approximately 45 minutes.

## 3. Measures

### 3.1. Primary Outcome

#### 3.1.1. The Brief Pain Inventory-Short Form (BPI-SF)

The primary outcome was pain (interference and severity) as measured by the BPI-SF. The BPI-SF, originally developed for cancer pain, measures both pain severity and pain interference [19] and has been shown to have strong psychometric properties, with Cronbach's alpha (*α*) ranging from 0.80 to 0.92 [19]. The BPI has been validated against pain visual analog scale oncology patients for measuring pain and shows a high correlation between the subscales of the BPI (*r* = 0.71, *p* < 0.01) [20].

### 3.2. Secondary Outcomes

#### 3.2.1. The Profile of Mood States-Short Form (POMS-SF)

The Profile of Mood States-Short Form (POMS-SF) is a measure of transient, distinct mood states, which calculates Total Mood Disturbance (TMD) using six subscales: tension, depression, fatigue, vigor, anger, and confusion [21]. Internal consistency estimates of the POMS-SF scales, using coefficient alpha, have ranged from 0.80 to 0.91 [22]. The POMS-SF has been frequently used in psychosocial oncology research with available norms [23] and validated by determining its ability to detect changes in distress scores in cancer patients [24].

#### 3.2.2. The Pittsburgh Sleep Quality Index (PSQI)

The Pittsburgh Sleep Quality Index (PSQI) is a 19-item questionnaire that measures the quality of sleep and sleep disturbances [25]. Measures of internal consistency have indicated the PSQI has a Cronbach's *α* of 0.83. Through the use of a contrasting approach, the PSQI has been validated by its ability to significantly differentiate between groups with low fatigue and high fatigue in the cancer population [26]. The PSQI also can differentiate poor and good sleepers against the benchmark of polysomnography, and it has clinical cutoff scores [25].

#### 3.2.3. The Functional Assessment of Cancer Therapy-Fatigue (FACT-F)

The Functional Assessment of Cancer Therapy-Fatigue (FACT-F) is comprised of the FACT-G [27], a 20-item questionnaire that measures physical, social/family, emotional, and functional well-being within the cancer population [28], plus an additional 13 questions related to fatigue [29]. Internal consistency for the entire FACT-F scale has been found to be between 0.95 and 0.96 [30]. The FACT-F subscale has also been shown as highly correlated to other measures of fatigue, such as the Piper Fatigue Scale [30], and responsive to fatigue treatment interventions [31].

#### 3.2.4. The Inventory of Socially Supportive Behaviors (ISSB)

The Inventory of Socially Supportive Behaviors (ISSB) Short Form is an 18-item questionnaire designed to measure social support, which asks participants to indicate the number of times they have experienced particular behaviors in the past month [32]. The inventory measures four different dimensions of social support: emotional and instrumental support, as well as directive and cognitive informational guidance. The ISSB questionnaire has been shown to have an internal consistency (Cronbach's *α*) of 0.84 [32] and a strong correlation (*F* (2, 178) = 6.54, *p* < 0.002, *R*^2^ = .069) between scores and the number of people respondents feel they can confide in [33].

## 4. Data Analysis

### 4.1. Sample Size

In order to determine if there was a difference between the standard treatment and the experimental treatment, a minimum of 62 patients (31 per group) was required to be 80% sure that the lower limit of a one-sided 95% confidence interval (or equivalently a 90% two-sided confidence interval) will be above the predetermined noninferiority limit of −1 [34]. The noninferiority limit was determined using standard deviation (SD) of the difference in change in pain interference from a previously published noninferiority trial examining chronic pain [35].

### 4.2. Statistical Analyses

Participants' clinical and demographic characteristics were described using descriptive statistics. Analyses were conducted as per intent-to-treat (ITT) principles, whereby all participants who were randomized and completed the baseline assessment were included. As ITT analyses can bias results toward equivalence [36], an additional analysis was conducted with the completer sample (those who completed the intervention per protocol) [36].

#### 4.2.1. Noninferiority Margins

The noninferiority margin for the BPI-inference subscale was set at 1 point for a number of reasons. First, evidence suggests that a 1-point change on the BPI interference scale signifies minimally clinically important change at the individual level [37]. Additionally, there are no specific recommendations for clinically important group differences for commonly used pain measures [38]. Given the mean differences for efficacious treatments at the group level are generally smaller than what is considered clinically meaningful at the individual level [38], and based on past studies [35], a 1-point change seemed appropriate [35, 38]. As there is little data available for clinically meaningful noninferiority margins for BPI severity or the secondary outcomes at the group level, for each of these, the noninferiority margin was set at 0.5 times the standard deviation (SD) (obtained by multiplying 0.5 to the pooled SD of raw change scores (baseline minus posttreatment)), a generally accepted rule-of-thumb for clinically meaningful change [34, 35].

#### 4.2.2. Assessment of Noninferiority

Modeling of linear mixed effects was used to examine the change in scores for primary and secondary outcomes across pre- and postintervention assessments [39]. Group (1 = individual AP; 2 = group AP), time (categorically coded), and the group∗time interaction were included as fixed effects. Random intercepts, as well as random slopes, were included as random effects [35]. The noninferiority of group AP with respect to individual AP delivery was assessed using a 2-sided 95% confidence interval (CI) on the difference in change rate from baseline to postintervention (i.e., the group^*∗*^time interaction coefficient) between arms (group minus individual) [29]. A positive value for the difference in change rate for BPI pain and interference scores indicated more reduction in the pre-post scores in the group AP compared with the individual AP. On measures for which lower postintervention scores were indicative of improvement, noninferiority was claimed if the lower bound of the 95% CI was greater than the noninferiority margin. Likewise, on measures for which higher postintervention scores were indicative of improvement (ISSB, FACT), noninferiority was claimed if the upper bound of the 95% CI was smaller than the noninferiority margin.

Within-group effect sizes were calculated using the Cohen *d* statistic and presented where appropriate to represent changes within groups. Per Cohen's guidelines, *d* = .2 is considered a small effect, *d* = .5 a medium effect, and *d* = .8 a large effect [34]. All statistical analyses were conducted using IBM SPSS version 23.0 (IBM Corp, Armonk, NY). The *p* value < 0.05 indicates statistical significance.

#### 4.2.3. Cost Assessment

Costs associated with the group and individual AP included the acupuncturist fee per session for both arms, as well as the cost of needles. Space in the treatment center was provided free of charge. The cost of the treatments (using a patient or payer perspective) was calculated by comparing the average cost of one treatment cycle (six weeks of treatment) for one group AP participant to the cost of one treatment cycle for an individual AP participant. Because not every group session was full, attendance for each group AP session was tracked, and the hourly cost of the practitioner was divided among this number in order to calculate the actual cost per participant per session and subsequently cost per treatment cycle.

## 5. Results

### 5.1. Sociodemographic and Clinical Characteristics

The mean age of participants was slightly higher in group AP (49.6 years) compared to individual AP (41 years) (*p*=0.007), due to a failure of randomization since we did not stratify on age. There were no differences in other sociodemographic baseline characteristics across the two arms. The majority of the sample in both arms was females and the majority of the participants were married or cohabitating. Only 1/4^th^ of the participants in individual AP and 1/3^rd^ in group AP were employed (part-time or full-time). The majority of participants (>75%) in both arms had a higher education (college, technical school, or university). Clinically, the majority of participants in both arms were breast cancer patients, and in the first three treatment cycles, the majority had had chemotherapy and/or surgery (Table 1).

The mean number of sessions attended by participants in individual AP (10 ± 3.68) and group AP (9.4 ± 3.82) was similar (*t* = .72, *p*=0.48). Overall attrition (participants who did not complete posttreatment assessment at the end of 12 sessions) was 30.7%. There were no statistically significant differences in attrition between the individual AP (20.5%) and group AP (40.0%) (Chi-square = 3.35; *p*=0.067) treatment arms. Patient characteristics for those who withdrew during treatment were not statistically different from those who completed treatment regarding sex, marital status, and educational status (Ps > 0.05). However, participants who were employed (part-time/full-time) were less likely to withdraw (*n* = 1, 5.3%), compared to those who were unemployed/retired or were unable to work due to disability (*n* = 18, 38.3%) (Chi-square 7.20, *p*=0.007). There were no significant differences in baseline BPI, PSQI, POMS-TMD, ISSB, and FACT scores between completers and noncompleters (Ps > 0.05). More participants in the individual AP (80%) completed both preassessment and postassessment than those in the group AP (58%) (Chi-square = 3.35, *p*=0.07).

### 5.2. Noninferiority Analysis

#### 5.2.1. Primary Outcome

**Figure 2:**
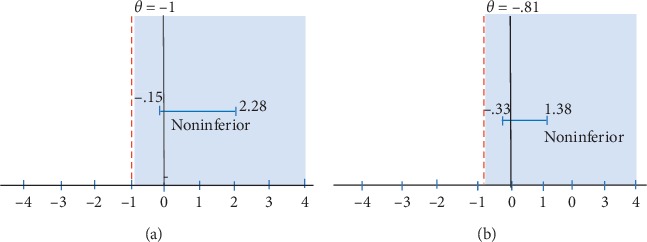
(a) Noninferiority plot for BPI interference. (b) Noninferiority plot for BPI severity.

**Figure 3:**
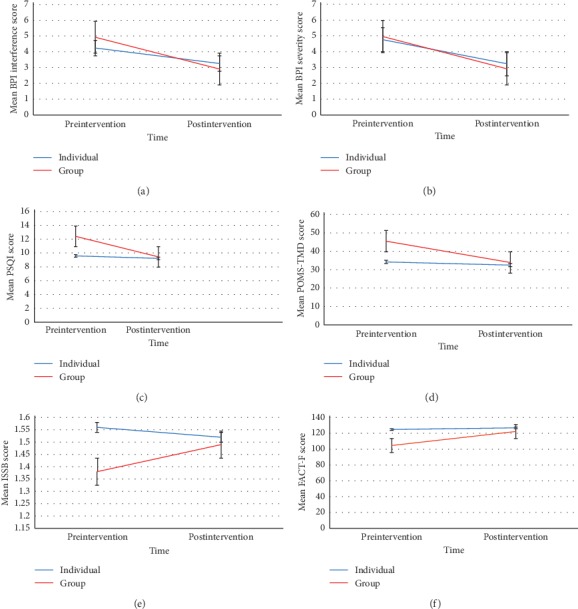
(a) Mean pre-post BPI interference scores for the group vs. individual acupuncture. (b) Mean pre-post BPI severity scores for the group vs. individual acupuncture. (c) Mean pre-post PSQI scores for the group vs. individual acupuncture. (d) Mean pre-post POMS-TMD scores for the group vs. individual acupuncture. (e) Mean pre-post ISSB scores for the group vs. individual acupuncture. (f) Mean pre-post FACT-Fatigue scores for the group vs. individual acupuncture.

Results from the noninferiority analysis for pain interference and severity are reported in Table 2. On the BPI interference scale, noninferiority of group versus individual AP was found, because the lower bound of the 2-sided 95% CI on differences in change rate between arms from baseline to posttreatment of 1.03 (−0.15–2.20) was greater than the noninferiority margin of −1 (Figure 2(a)). There were no differences between the ITT sample (*n* = 74) and the completer sample (*n* = 54). Noninferiority was met for both BPI interference physical and BPI interference psychological subscales. Similarly, for BPI severity, noninferiority was met because the lower bound of the 2-sided 95% CI on differences in change rate of 0.52 (−0.33–1.38) was greater than the noninferiority margin of −0.81 (Figure 2(b)). We found nonsignificant effects for group and the group^*∗*^time interaction, and the noninferiority analyses were based on these models. Figures 3(a) and 3(b) illustrate that there was no significant difference in reduction in pain interference and severity between group AP and individual AP.

#### 5.2.2. Secondary Outcomes

Noninferiority of group AP compared with individual AP was also met for all the secondary outcomes (Table 3). For PSQI [2.60 (0.33–4.88)] and POMS-TMD [9.86 (0.85–18.86)], the lower bound of the 2-sided 95% CI on differences in change rate was greater than the noninferiority margins of −1.65 and −7.52, respectively. For ISSB [−0.15 (−0.42–0.13)] and FACT-F [−15.57 (−25.60–5.54)], the upper bound of the 95% CI was smaller than noninferiority margins of 0.26 and 8.54, respectively. Results from linear mixed effects models also revealed significant group^*∗*^time interactions for all secondary outcomes with the exception of ISSB. Specifically, compared with the individual intervention, the change in POMS-TMD, PSQI, and FACT scores from baseline to postintervention was higher for the group intervention, showing that the group intervention exhibited greater improvement on these outcomes compared with the individual intervention. Figures 3(c) and 3(d) illustrate that there was a greater reduction in PSQI and POMS-TMD scores in group AP compared to individual AP.  Figure 3(e) illustrates that there was no significant difference in change in pre-post ISSB scores between GA and IA.  Figure 3(f) shows that there was a greater improvement in FACT-F scores in GA compared to IA.

Results for primary and secondary outcomes for the per-protocol analysis (completer sample) are provided in online Appendices 1 and 2 but did not differ substantively from the ITT analyses.

#### 5.2.3. Cost Analysis

The actual costs for group AP were nearly half those of individual AP. The acupuncturist's fee was set at $50 per hour (discounted for the study; a usual fee is $100). Thus, for individual AP, the cost of a 45-minute session per participant was $37.5, and for group AP, the cost of a 90-minute session for each group with an average of 4 participants per group was $75, amounting to $18.75/person. For individual AP, the total mean cost of administering AP per participant was $395. This included the mean cost of administering AP ($375) for the average of 10 sessions and the mean cost of needles ($20) per person (total cost = $37.5 per session × 10 sessions + $20 for needles). Likewise, for group AP, the total mean cost of administering AP was $196.25. This included the mean cost of administering AP for an average of 10 sessions ($176.25) and the mean cost of needles ($20) per person (total cost= ($75 per session/4 × 9.4 + $20 for needles). The cost of the 30-minute assessments at initial intake was similar for all participants at the rate of $25 per participant regardless of group. Therefore, the total mean cost for participants in individual AP was $420 and, for those in group AP, was $221.25.

## 6. Discussion

This is the first study to examine the noninferiority of group AP compared to individual A, the gold standard, in a prospective randomized trial. These findings provide strong evidence in support of the hypotheses that group-based AP is both statistically and clinically noninferior and in fact superior on many outcomes when compared to individualized treatment for cancer pain and can be delivered at half the cost. Group AP produced significant reductions in pain interference and severity, compared with those of individual AP at posttreatment measurements. Moreover, group AP was superior to individual AP for improving the psychological component of pain interference, sleep quality, distress, and fatigue. Contrary to our expectation that group AP would have a greater impact on perceived social support as patients would be able to bond better in the group setting, both interventions were similar in terms of their impact on perceived social support.

These findings are consistent with other studies that support the efficacy of group AP for treating pain in other conditions as well as for cancer pain [40]. For example, Tofthagen et al. [13] reported significant improvement in cancer-related pain and numbness for patients undergoing group AP; however, they used a retrospective study design and had no comparison group. Similarly, in a qualitative study, Chuang et al. [12] reported that patient experiences in group AP were on par with individual AP for pain relief and improvement in QoL; however, their findings were limited to patients' perceived value and treatment experiences in individual versus group AP, rather than objective changes in health outcomes over time through standardized measures. Kligler et al. [14] reported on the effectiveness of group AP for reducing pain severity and interference as well as symptoms of depression in primary care patients experiencing chronic neck, back, or shoulder pain or osteoarthritis. However, since they did not have a control group, they could not directly attribute improvements to AP therapy. The effect sizes of improvement in patient scores in group AP were large for pain interference and severity, sleep, fatigue, and psychological distress, compared to other studies of individual AP in similar cancer samples [41–43]. Using a strong noninferiority RCT design in our study to overcome these limitations of past work, our findings not only lend support for the comparative effectiveness of group AP but also demonstrate its cost-effectiveness.

### 6.1. Limitations

This study is characterized by several strengths, including random assignment, use of a prospective study design, an active comparison group, adequate power to test our hypotheses, and a similar rate of attrition between the two groups. However, there are also several limitations. First, attrition was relatively high particularly in group AP arm; it would be important in future work to understand why 31% of the participants were unable to, or chose not to, complete the full course of the AP treatments. Furthermore, the majority of the participants across both arms were females, mostly breast cancer patients, mostly white, and mostly highly educated. The study needs to be replicated in other types of patients with diverse cancer diagnoses and with homogenous pain conditions and belonging to culturally diverse populations and socioeconomic conditions. The findings also need to be replicated in large samples to confirm the reliability and validity. Additionally, the study did not have a ‘no treatment' control group, as we already knew from previous research that individual AP was likely better than no treatment/usual care, and for both ethical and recruitment reasons, we did not wish to offer waitlist or usual care without AP. Additionally, participants were not blinded to treatment and there was no sham AP condition. These limitations allow for effects on outcomes due to the psychological expectancy of benefit (placebo), which could have impacted our results. This study was meant to be pragmatic, offering AP as in real-world settings, which never deploy sham or blinded AP, so these effects are likely to mirror real-world outcomes.

Lastly, clinically meaningful noninferiority margins at the group level have not been established for many of the secondary outcomes and we had to rely on statistically derived noninferiority margins of 0.5 SDs, which may have been either too conservative or too generous to infer noninferiority. However, this limitation was offset to an extent as group AP turned out to be superior to individual AP on many of the secondary outcomes using conventional statistical group comparisons.

## 7. Conclusions

Biweekly AP therapy offered in the group setting for 6 weeks, delivered for half the cost of individual AP, was as effective as individual AP for reducing cancer-related pain interference and severity and was superior to individual AP in improving sleep quality, fatigue, and psychological distress. Group AP may be an effective treatment option for patients who may otherwise be unable to afford it due to relatively high costs and the lack of universal coverage for AP treatment. Where possible, cancer centers and practitioners should consider offering AP in group-based settings, rather than individually, for routine cancer pain treatments as a more cost-effective delivery model.

## Figures and Tables

**Table 1:** Demographic and clinical characteristics of participants in individual vs. group acupuncture interventions.

Characteristic	Individual AP	Group AP
Age at joining (*N* = 70) (mean) (SD)	40.87 (33.03)	49.59 (21.50)

Sex		
Females	29 (76.3%)	30 (88.2)

Employment status		
Disability/unemployed/retired	28 (75%)	21 (67.7%)
Employed (PT/FT)	8 (25%)	10 (32.3%)

Highest level of education		
Up to high school	9 (23.7%)	5 (16.7%)
College/technical school/some university	16 (42.1%)	14 (46.6%)
Masters/postgrad degree/doctoral	13 (34.2%)	11 (36.7%)

Marital status		
Single/divorced/widowed	9 (25%)	8 (26.6%)
Married/cohabitating	27 (75.0%)	22 (73.3%)
Cancer type		
Breast	19 (48.7%)	21 (61.8%)
Gastrointestinal	6 (15.4%)	6 (17.6%)
Gynecological	4 (10.3%)	3 (8.8%)
Hematological	5 (12.8%)	2 (5.9%)
Skin	1 (2.6%)	1 (2.9%)
Lung	3 (7.7%)	1 (2.9%)
Head and neck	1 (2.6%)	0 (0.0%)

Treatment type		
Treatment 1 (*N* = 72)		
Chemotherapy	15 (39%)	17 (52%)
Radiotherapy	7 (18%)	3 (9%)
Surgery	14 (36%)	10 (30%)
Hormonal therapy	2 (5%)	3 (9%)
Treatment 2 (*N* = 54)		
Treatment 2 (*N* = 54)	14 (48%)	9 (36%)
Chemotherapy	4 (14%)	6 (24%)
Surgery	9 (31%)	9 (36%)
Bone marrow transplant	1 (3.4%)	0 (0%)
Immunotherapy	1 (3.4%)	0 (0%)
Stem cell	0 (0%)	1 (4%)
Treatment 3 (*N* = 26)		
Chemotherapy	1 (9%)	2 (13%)
Radiotherapy	3 (27%)	5 (33%)
Surgery	4 (36%)	1 (7%)
Hormonal therapy	3 (27%)	7 (47%)
Treatment 4 (*n* = 6)		
Radiotherapy	1 (25%)	1 (50%)
Surgery	0 (0%)	1 (50%)
Hormonal therapy	3 (75)	0 (0)

**Table 2:** Mean and SE of primary outcomes obtained from linear mixed effects models and the noninferiority margins and difference in change rate from baseline to posttreatment (ITT sample).

Outcome	Intervention	Time-point	Mean (SE)	Noninferiority margin	Change^*∗*^	Effect size (cohen *D*)	Group^*∗*^time *F*, *p* value (sig)	Difference in change (95% CI) (group-individual)
*BPI interference*	Individual	Pre	4.24 (0.34)	−1	0.98	0.48	3.02, *p*=0.08	1.03 (−0.15–2.20)
		Post	3.26 (0.34)					
	Group	Pre	4.92 (0.37)		2.01	1.06		
		Post	2.91 (0.43)					

*BPI interference- PHYSICAL*	Individual	Pre	4.49 (0.40)	−1.27	1.04	0.44	0.73, *p*=0.39	0.56 (−0.76–1.90)
		Post	3.45 (0.43)					
	Group	Pre	4.89 (0.43)		1.60	0.70		
		Post	3.29 (0.49)					

*BPI interference- psychological*	Individual	Pre	4.01 (0.34)	−1.14	0.91	0.46	5.22, *p*=0.03	1.39 (0.17–2.60)
		Post	3.10 (0.37)					
	Group	Pre	4.93 (0.37)		2.30	1.29		
		Post	2.63 (0.43)					

*BPI severity*	Individual	Pre	4.76 (0.27)	−0.81	1.52	0.89	1.51, *p*=0.22	0.52 (−0.33–1.38)
		Post	3.24 (0.29)					
	Group	Pre	4.96 (0.29)		2.04	1.29		
		Post	2.92 (0.34)					

^*∗*^A positive value indicates a change in the desired direction or reduction in pain scores from baseline to postintervention.

**Table 3:** Mean and SE of secondary outcomes obtained from linear mixed effects models and the noninferiority margins and difference in change rate from baseline to posttreatment (ITT sample).

Outcome	Intervention	Time-point	Mean (SE)	Change^*∗*^(pre-post)	Effect size (cohen *D*)	Group^*∗*^time *F*, *p* value (sig)	Noninferiority margin	Difference in change (95% CI)
PSQI global	Individual	Pre	9.58 (0.73)	0.36	0.19	5.39, *p*=0.03	−1.65	2.60 (0.33–4.88)
		Post	9.22 (0.82)					
	Group	Pre	12.40 (0.74)	2.96	0.66			
		Post	9.44 (0.84)					

POMS-TMD	Individual	Pre	34.22 (2.93)	1.72	0.05	4.87, *p*=0.03	−7.52	9.86 (0.85–18.86)
		Post	32.50 (3.09)					
	Group	Pre	45.51 (3.14)	11.61	0.82			
		Post	33.93 (3.56)					

ISSB (average)	Individual	Pre	1.56 (0.12)	0.04	0.01	1.10, *p*=0.29	0.26	−0.15 (−0.42–0.13)
		Post	1.52 (0.12)					
	Group	Pre	1.38 (0.13)	−0.52	0.01			
		Post	1.49 (0.14)					

FACT-F	Individual	Pre	124.88 (4.90)	−2.1	0.12	9.76, *p*=0.003	8.54	−15.57 (−25.60–5.54)
		Post	126.98 (4.91)					
	Group	Pre	104.68 (5.06)	−17.67	0.86			
		Post	122.35 (5.27)					

^*∗*^Higher scores on BPI, PSQI, and POMS indicate worse pain, sleep, and mood, respectively. A positive value indicates a change in the desired direction or reduction in pain scores from baseline to postintervention. ^*∗*^Higher scores on ISSB and FACIT indicate better social support and QoL, respectively. A negative value indicates a change in the desired direction or improvement in scores from baseline to postintervention.

**Table 4:** 

Outcome	Intervention	Time	Mean (SE)	Noninferiority margin	Difference in change (95% CI)
BPI interference	Group	Pre	4.75 (0.45)	−1	
		Post	3.20 (0.50)		
	Individual	Pre	4.23 (0.41)		0.72 (−0.67–2.11)
		Post	3.40 (0.44)		

BPI interference-PHY	Group	Pre	4.58	−1.27	0.77 (−0.71–2.25)
		Post	2.87		
	Individual	Pre	4.35		
		Post	3.40		

BPI interference-PSYCH	Group	Pre	4.95	−1.14	1.76 (0.45–3.06)
		Post	2.45		
	Individual	Pre	3.78		
		Post	3.04		

BPI severity	Group	Pre	4.83 (0.32)	−0.81	0.45 (−0.55–1.46)
		Post	3.06 (0.35)		
	Individual	Pre	4.58 (0.31)		
		Post	3.26 (0.32)		

**Table 5:** 

Outcome	Time point	Intervention	Mean (SE)	Noninferiority margin	Difference in change (95% CI)
PSQI global	Group	Pre	12.37 (.91)	1.65	2.77 (0.18–5.35)
		Post	9.99 (.98)		
	Individual	Pre	9.17 (1.01)		
		Post	9.56 (1.08)		

POMS-TMD	Group	Pre	42.04 (3.79)	7.52	3.54 (−5.98–13.07)
		Post	36.23(4.07)		
	Individual	Pre	34.87 (3.72)		
		Post	32.61 (3.76)		

ISSB	Group	Pre	1.38 (.15)	.26	−0.19 (−0.53–0.13)
		Post	1.48 (.16)		
	Individual	Pre	1.62 (.14)		
		Post	1.52 (.15)		

FACT-F	Group	Pre	110.67 (5.92)	8.54	−10.85 (−22.38–0.68)
		Post	123.08 (6.54)		
	Individual	Pre	121.45 (5.78)		
		Post	123.02 (6.27)		

## Data Availability

The datasets used and/or analyzed during the current study are available from the corresponding author on reasonable request.

## Appendix

### A. Mean and SE of Primary Outcomes Obtained from Linear Mixed Effects Models and the Noninferiority Margins and Difference in Change Rate from Baseline to Posttreatment (Completer Sample)

The results of the mean and SE of primary outcomes are given in Table 4.

### B. Mean and SE of Secondary Outcomes Obtained from Linear Mixed Effects Models and the Noninferiority Margins and Difference in Change Rate from Baseline to Posttreatment (Completer Sample)

The results of the mean and SE of primary outcomes are given in Table 5.

## Abbreviations

AP: Acupuncture
SD: Standard deviation
TCM: Traditional Chinese Medicine
BPI: Brief Pain Inventory
PSQI: Pittsburgh Sleep Quality Index
POMS-TMD: Profile of Mood States-Total Mood Disorder
ISSB: Inventory of social supportive behaviors
FACT-F: Functional Assessment of Cancer Therapy-Fatigue.
